# Microwave-assisted synthesis and characterization of Xanthan gum-grafted polyacrylamide hydrogel for the removal of acid red 8 dye from aqueous solutions

**DOI:** 10.1038/s41598-025-14539-2

**Published:** 2025-09-12

**Authors:** Eman Aly Fadl, Wagih Abdel Alim Sadik, Abdel Ghaffar El Demerdash, Hanan Adel Moukhtar, Marwa El-Sayed Hamza

**Affiliations:** Department of Materials Science, Institute of Graduate Studies & Research, Alexandria University, Alexandria, Egypt

**Keywords:** Hydrogel, Xanthan gum, Acrylamide, Thermodynamics, Kinetics model, Recyclability, Chemistry, Materials science

## Abstract

This study focuses on creating a sustainable, biodegradable, and affordable hydrogel from xanthan gum grafted polyacrylamide using a microwave-assisted synthesis method. The hydrogel is designed to remove Acid red 8 dye (AR8) from aqueous solutions using adsorption technique. Characterization was done using FTIR, SEM, and TGA analyses. Factors affecting the adsorption of AR8 dye onto the hydrogel such as grafting percent, pH, hydrogel dosage, contact time and the dye’s initial level in solution, temperature, as well as the reusability studies were investigated. The results indicated that at pH 1, the swelling ratio reached 1720% after 20 min. The maximum adsorption capacity of AR8 dye was 177 mg/g after 20 min, with no significant increase beyond 20 min. The percent of dye removal (%) increased from 84.7 to 88.6 with increasing the hydrogel dosage from 0.0063 to 0.05 g/L and there was no increase above 0.05 g/L. The percent of dye removal of AR8 increased with increasing the percentage of grafting due to rising the number of functional groups grafted on XG backbone. Also, q_e_ decreased as temperature increased from 20 to 60 °C.

**Supplementary Information:**

The online version contains supplementary material available at 10.1038/s41598-025-14539-2.

## Introduction

Water is the foundation of life, a fundamental resource that sustains all living organisms on earth. Its importance permeates every aspect of existence, from nourishing ecosystems to sustaining human health and powering industrial processes. However, this vital resource faces a myriad of threats, one of which is the pollution caused by dyes^1,2^. Dyes are widely utilized across numerous industries, including textiles, packaging, and plastics. possess vibrant colors that enhance products’ appeal. Yet, their disposal into water bodies poses significant environmental challenges. When dyes leach into waterways, they not only compromise water quality but also disrupt aquatic ecosystems, affecting the flora and fauna within them. Understanding the relationship between water and dye pollutants underscores the critical need for sustainable practices in industrial processes^3,4^.

Hydrogels offer a potential solution for dye removal from water due to their exceptional properties and versatility. These three-dimensional networks of polymer chains are highly absorbent and can hold large volumes of water, making them ideal for capturing dye molecules from aqueous solutions^5^. One of the key advantages of using hydrogels for dye removal is their ability to maintain stability and integrity even after multiple cycles of adsorption and desorption. This makes them cost-effective and sustainable alternatives to traditional methods of dye removal, such as activated carbon or membrane filtration. Xanthan gum (XG) is a natural polysaccharide that is produced through fermentation by the bacterium Xanthomonas campestris^6,7^. Similar to polysaccharides, XG is biodegradable, biocompatible, non-toxic and cost effective^8^.

**Fig. 1:**
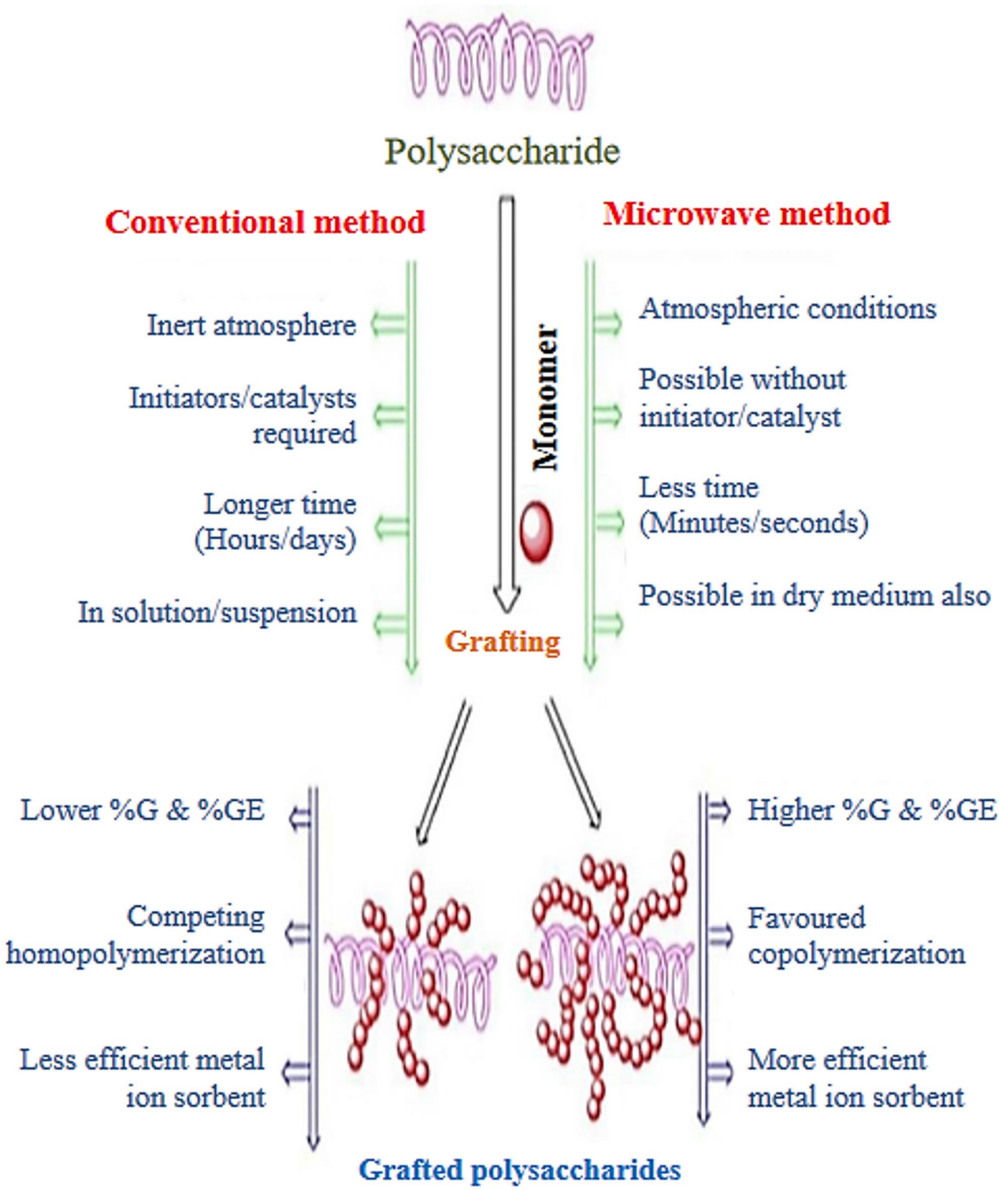
Microwave induced grafting of polysaccharides versus conventional grafting method.

Microwave-assisted grafting of polysaccharides involves using microwave irradiation to facilitate the attachment of functional groups or polymers onto the polysaccharide backbone. This method offers advantages such as shorter reaction times, higher yields, and improved control over the grafting process compared to conventional methods^9^ such as (Ecreg et al.^10^, prepared “poly acrylamide-co-acrylic acid” hydrogels via free-radical polymerization using conventional and microwave heating. The conventional approach required 90 min at 60 degrees Celsius (^o^C), whereas microwave synthesis took only 2 min, saving energy, time and reaction components) and (Etemadi Moghaddam, H., & Baghban Salehi, M^11^ synthesized multi-network (MN) self-healing hydrogels using xanthan gum (XG) through conventional and microwave-assisted methods via free-radical polymerization. The synthesis materials included XG, acrylamide, acrylonitrile, sodium dodecyl sulfate, ferric chloride hexahydrate, and ammonium persulfate. Conventional synthesis took 60 min at 70 °C, while microwave synthesis was completed in 4.5 min at 540 W, enhancing energy and material efficiency). The difference between conventional and microwave assisted methods is mentioned in Fig. 1.

Our research stands out as it aims to create a cost-effective, fast synthesis (few seconds) and efficient Xanthan gum-g-poly acrylamide (XG-g-PAm) hydrogel which synthesized from biodegradable polysaccharide (xanthan gum) when degraded, the non toxic by product are not affecting the environment, using microwave-assisted grafting and to evaluate its effectiveness in removing anionic acid red 8 (AR8) dye from water through a single batch adsorption method. We selected AR8 dye for this study due to its carcinogenic nature, commonly found in wastewater from textile manufacturing and leather processing industries, posing risks to both ecosystem and human health by discoloring water and depleting oxygen levels^12–14^.

In this study, different grafting ratios of xanthan gum with acrylamide were prepared to obtain a hydrogel with high efficiency for the removal of Acid Red 8 dye. As shown in Table 5, the hydrogel exhibited superior maximum adsorption capacity compared to previously reported studies.

## Experimental work

### Materials

Xanthan gum polysaccharide (XG) (C_35_H_49_O_29_, molecular weight 933.748 g/mol and biological source is sugar fermented by xanthomonas campestris bacteria), Acid red 8 dye (AR8) (C₁₅H₁₄N₃NaO₅S₂, molecular weight 480.42 g/mol and maximum wavelength = 508 nm), hydrochloric acid (HCL), and sodium hydroxide (NaOH) were purchased from Chemajet, Egypt, and acrylamide (Am) (C₃H₅NO, molecular weight 71.08 g/mol and assay of 98%) was supplied from Loba Chemie, Mumbai- India, and used without further purification. Potassium persulfate (KPS) (K_2_S_2_O_8_,99%) was purchased from PIOCHEM, Egypt.

### Preparation of Xanthan gum-g-polyacrylamide hydrogel

Different quantities of acrylamide (1, 2.5, 5, 7.5, 10 g) were dissolved in distilled water. Potassium persulfate (KPS) (0.3 g) was added as an initiator, and N, N’-methylene-bis-acrylamide (MBA) (0.1% of the total weight) was added with continuous stirring vigorously. Once dissolved, 1 gram of XG powder was added and stirred vigorously for at least one hour to achieve a uniform solution. The reaction vessel was then placed in a microwave oven at 800 W (~ 65 °C) and then cooled using an ice bath. to avoid unwanted homopolymerization reactions. The microwave irradiation and cooling cycles were conducted for a maximum of 2 min until a thick gel-like material formed^15,16^.

**Fig. 2:**
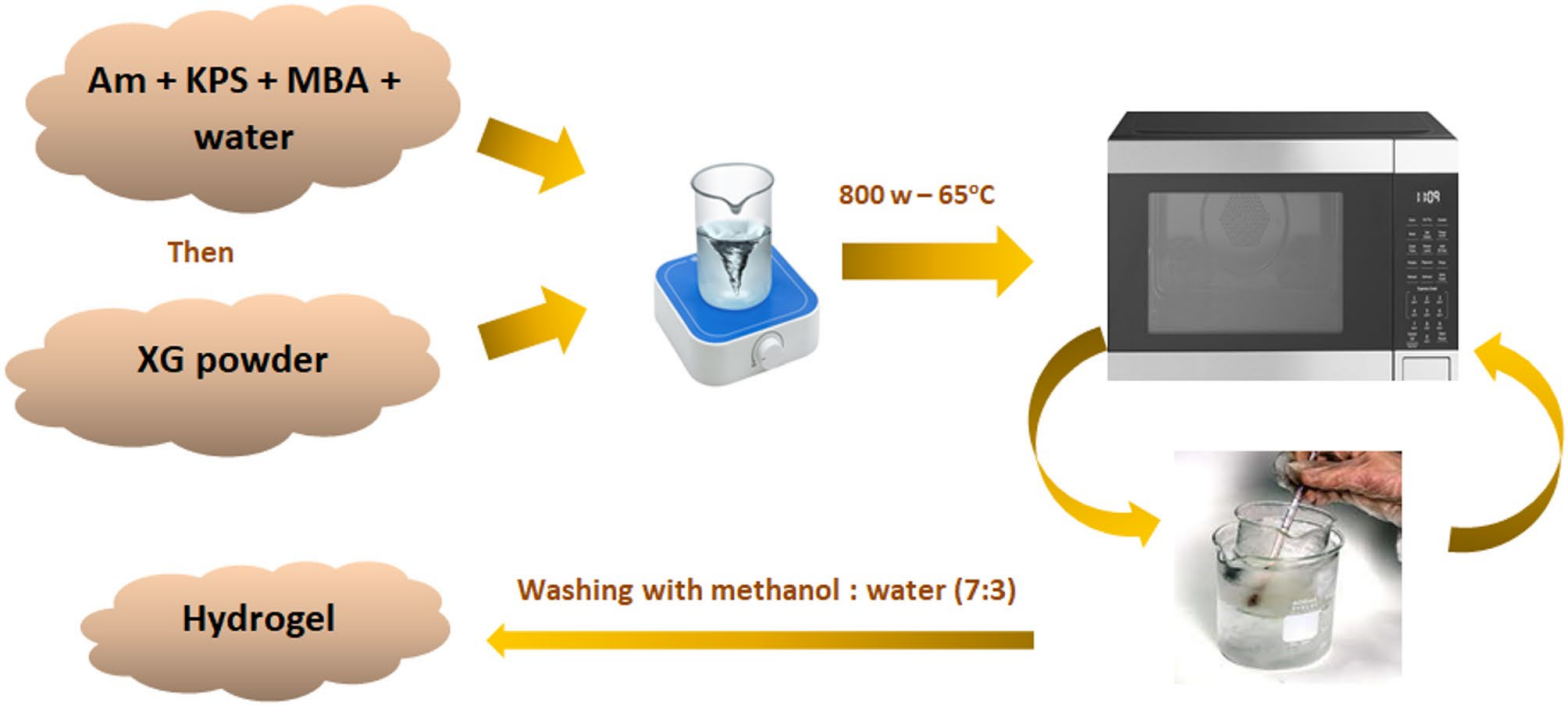
Microwave-assisted synthesis of XG-g-PAm hydrogel.

Once the microwave irradiation process was finished, the container and its contents were allowed to cool. The next step involved washing with a methanol and water mixture (7:3 v/v) to eliminate any unreacted components and homopolymer. The synthesized hydrogel was then cut into tiny pieces, dried at 60 °C until reaching a constant weight, and ground into a fine powder. Figure 2 illustrated the synthesis process, and Table 1 listed the amounts used in the fabrication of different grades of XG-g-PAm hydrogel.

**Table 1:** Different grades of synthesized XG-g-PAm hydrogel.

Grade	XG (W_0_)		Am (W_2_)		MBA		KPS		Hydrogel weight (g) (W_1_)	%G	%GE
	Wt. (g)	%	Wt. (g)	%	Wt. (g)	%	Wt. (g)	%			
XG-g-PAm 1	1	43.4	1	43.4	0.0023	0.1	0.3	13.0	2.3023	65.5	65.5
XG-g-PAm 2	1	26.3	2.5	65.7	0.0038	0.1	0.3	7.9	3.8038	226.3	90.5
XG-g-PAm 3	1	15.9	5	79.3	0.0063	0.1	0.3	4.8	6.3063	466.9	93.4
XG-g-PAm 4	1	11.4	7.5	85.1	0.0088	0.1	0.3	3.4	8.8088	714.3	95.2
XG-g-PAm 5	1	8.8	10	88.4	0.0113	0.1	0.3	2.7	11.3113	966.3	96.6

XG-g-PAm 4 was the optimum composition that used for all experiments and analysis.

### The grafting percentage (%G) and grafting efficiency (%GE)

%G and %GE of the XG-g-PAm hydrogel synthesized via microwave were assessed using the subsequent equations:

1 $${\%}\varvec{G}=\left(\frac{{\varvec{W}}_{1}-{\varvec{W}}_{0}}{{\varvec{W}}_{0}}\right)\times100$$

2 $${\%}\:\varvec{G}\varvec{E}=\left(\frac{{\varvec{W}}_{1}-{\varvec{W}}_{0}}{{\varvec{W}}_{2}}\right)\times100$$

W_0_, W_1_, and W_2_ represent the weight of XG, XG-g-PAm hydrogel and Am, respectively.

## Characterization of XG-g-PAm 4 hydrogel

### Fourier transforms infrared spectroscopy (FTIR)

XG, Am monomers and XG-g-PAm 4 hydrogel were characterized by FTIR in the range of 4000–450 cm^−1^, in solid state using KBr pellets. The samples were pulverized in a mortar and homogenized with KBr followed by pressing the mixture in a hydraulic press to cast the pellets into thin discs, using spectrophotometer (Perkin Elmer Spectrum BX, USA).

### Scanning electron microscopy (SEM)

The fundamental physical and surface morphology of XG-g-PAm 4 hydrogel and native XG sample were characterized by using SEM of JEOL model JSM-6010 LV (Japan). The specimens in the form of films were mounted on the specimen stabs and coated with thin film of gold by the ion sputtering electrode to increase the conductivity of the electron beam, then placed on equipment’s holder. All the equipment parameters like accelerated voltage, focusing, working distance, etc. were adjusted for every sample to obtain images with high resolution.

### Thermogravimetric analysis (TGA)

TGA was performed using thermal analyzer (SDTQ600, TA instruments, USA) in a nitrogen atmosphere; samples of approximately 6 mg were heated in an aluminum cell from ambient temperature to 600 °C with a heating rate of 10 °C/min.

### XG-g-PAm hydrogel synthesis mechanism

**Fig. 3:**
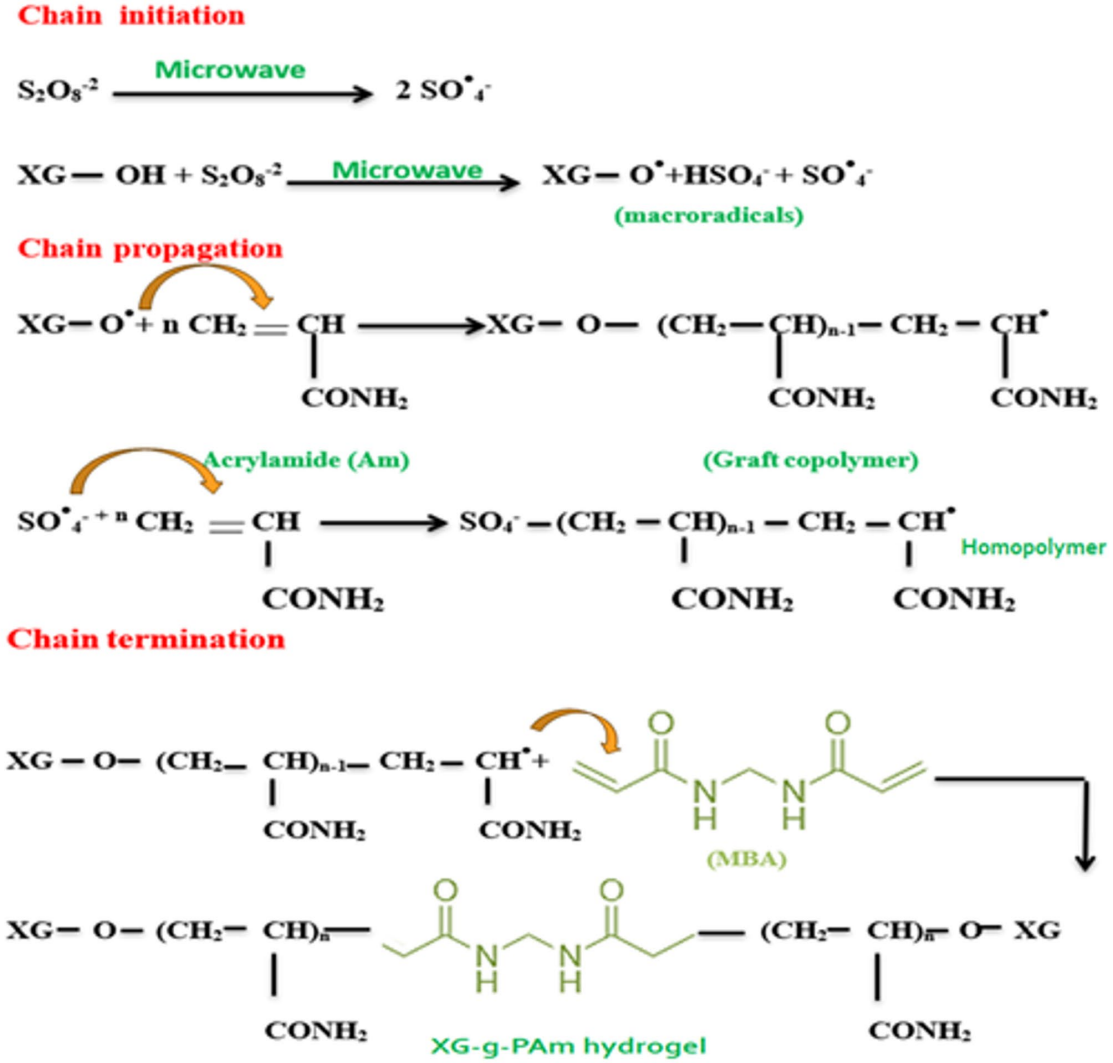
Proposed mechanism for formation of XG-g-PAm hydrogel.

An envisioned process for the production of XG-g-PAm hydrogel is outlined in Fig. 3, proposing a potential pathway for its synthesis.

It is a chain reaction mechanism that includes three major steps: initiation propagation and termination^17,18^.

### Determination of swelling ratio of prepared hydrogel

0.1 g of XG-g-PAm 4 hydrogel was immersed in 100 mL of distilled water for 48 h to reach equilibrium, which means that absorption stops. This allowed researchers to study the swelling of XG-g-PAm hydrogel. Subsequently, the engorged hydrogel was taken out from the beaker, and the surplus water was removed by mild shacking using a mesh. After the samples were reweighed, Eq. 3. could be used to determine the swelling percentage^19,20^.

3 $$\:\varvec{P}\varvec{e}\varvec{r}\varvec{c}\varvec{e}\varvec{n}\varvec{t}\:\varvec{o}\varvec{f}\:\varvec{s}\varvec{w}\varvec{e}\varvec{l}\varvec{l}\varvec{i}\varvec{n}\varvec{g}\:\varvec{r}\varvec{a}\varvec{t}\varvec{i}\varvec{o}\:\left({\%}\varvec{S}\varvec{R}\right)=\frac{{\varvec{W}}_{\varvec{t}}-{\varvec{W}}_{\varvec{d}}}{{\varvec{W}}_{\varvec{d}}}\times100$$

Where W_t_ and W_d_ represent the masses of the hydrogel in its swollen state at a specific time and in its dry state, respectively.

### Determination of point of zero charge (PZC)

PZC is the pH value at which a surface of XG-g-PAm hydrogel has a neutral overall electrical charge. It was investigated by pH drift equilibrium technique^20,21^. The pH value of 0.01 M NaCl solution was changed from 1 to 10.Then, 50 mg of XG-g-PAm 4 hydrogel was added and after 24 h pH stability was reached and final pH was measured^22^. To determine PZC, plots of the pH_initial_ against pH_initial−final_ values were established and the point of intersection is the pH_pzc_.

### Adsorption analysis of AR8 on XG-g-PAm hydrogel

The impact of different factors such as dose ranging from 0.0063 to 0.4 g/l, concentration between 100 and 3000 mg/l, pH ranging from 1 to 10, time from 0 to 60 min and %G on the adsorption of AR8 by XG-g-PAm hydrogel was examined under room temperature conditions. While analyzing each factor, all other variables were held constant. AR8 aqueous solution (100 mL) was poured into 250-mL flasks and placed on a rotary shaker (150 rpm) to ensure adequate contact time for equilibrium between XG-g-PAm hydrogel and dye solution. The absorbance of samples was monitored at regular intervals using a UV–Vis spectrophotometer at λ_max_ = 508 nm to assess the residual concentration of AR8 dye. The equilibrium amount of AR8 dye absorbed and its removal percentage were then calculated using the following equations:

4 $$\:{\varvec{q}}_{\varvec{e}}=\left({\varvec{C}}_{\varvec{i}}-{\varvec{C}}_{\varvec{e}}\right)\times\:\frac{\varvec{v}}{\varvec{w}}$$

5 $$\:\varvec{R}\varvec{e}\varvec{m}\varvec{o}\varvec{v}\varvec{a}\varvec{l}\:{\%}=\left(\frac{{\varvec{C}}_{\varvec{i}}-{\varvec{C}}_{\varvec{e}}}{{\varvec{C}}_{\varvec{i}}}\right)\times\:100$$

Here, q_e​_ represents the concentration of AR8 dye adsorbed onto the hydrogel at equilibrium (mg/g), C_i_​ is the initial concentration of AR8 dye in the solution (mg/L), C_e_​ is the equilibrium concentration of AR8 dye in the liquid phase (mg/L), v is the volume of AR8 dye solution (L) and w is the mass of XG-g-PAm hydrogel (g)^23,25^.

Three parallel experiments were made, and results of all experiments represent the mean of them.

### Adsorption kinetics

Adsorption kinetics is important for comprehending the mechanism of dye adsorption onto XG-g-PAm 4 hydrogel, as it helps describe the interactions between the adsorbate and adsorbent. To assess the adsorption kinetics, pseudo-first-order (Eq. 6) and pseudo-second-order (Eq. 7) kinetic models were employed, as shown below:

The pseudo-first-order kinetic model:

6 $$\:\varvec{l}\varvec{o}\varvec{g}\left({\varvec{q}}_{\varvec{e}}-{\varvec{q}}_{\varvec{t}}\right)=\varvec{l}\varvec{o}\varvec{g}\:{\varvec{q}}_{\varvec{e}}-\frac{{\varvec{K}}_{1}}{2.303}\varvec{t}$$

The pseudo-second-order kinetic model:

7 $$\:\frac{\mathbf{t}}{{\varvec{q}}_{\varvec{t}}}=\frac{1}{{{\varvec{K}}_{2}{\varvec{q}}_{\varvec{e}\:}}^{2}}+\frac{\mathbf{t}}{{\varvec{q}}_{\varvec{e}}}$$

where q_e_ and q_t_ (mg/g) represent the quantities of dye adsorbed on the hydrogel at equilibrium and at time t (min), respectively. k_1_ (min^−1^) or k_2_ (g/mg.min) values are the kinetic rate constants.

### Adsorption isotherm

The analysis of adsorption isotherms is essential for comprehending the interaction between the adsorbent and the adsorbate. In general the isotherm is the equation that relates the amount of adsorbate adsorbed on the adsorbent to the remaining concentration of the adsorbate in the solution. To investigate the adsorption process. Two widely recognized isotherm models, Langmuir (Eq. 8) and Freundlich (Eq. 9), were applied, as shown below:

8 $$\:\frac{1}{{\mathbf{q}}_{\mathbf{e}}}=\left(\frac{1}{{\mathbf{K}}_{\mathbf{L}}{\mathbf{Q}}_{0}}\right)\frac{1}{{\mathbf{C}}_{\mathbf{e}}}+\frac{1}{{\mathbf{Q}}_{0}}$$

9 $$\:{\varvec{q}}_{\varvec{e}}={\varvec{K}}_{\varvec{f}}{{\varvec{C}}_{\varvec{e}}}^{\frac{1}{\varvec{n}}}$$

Where C_e_ (mg/L) is the concentration of dye, q_e_ (mg/g) is the amount of dye adsorbed at equilibrium, q_m_ (mg/g) is the maximum adsorption capacity, K_L_ (L/g) is the Langmuir equilibrium constant, K_F_ (L/g) is the Freundlich isotherm constant and *1/n* is the adsorption intensity.

### Adsorption thermodynamics

Information about the type and mechanism of the adsorption process can be provided by thermodynamic parameters including (∆H), (∆G), and (∆S) of the adsorption process. They were measured using the following Eqs. (10, 11, 12, 13)^26–28^:

10 $$\Delta{G}=-\mathbf{RT}\mathbf{ln}\mathbf{K}_{d}$$

11 $$\:{\varvec{K}}_{\varvec{d}}\:=\frac{{\varvec{C}}_{\varvec{a}\varvec{d}\varvec{s}}}{{\varvec{C}}_{\varvec{e}}}=\frac{{\varvec{C}}_{\varvec{o}}-{\varvec{C}}_{\varvec{e}}}{{\varvec{C}}_{\varvec{e}}}$$

12 $$\Delta{G}=\Delta{H}-T\Delta{S}$$

13 $$\:{\varvec{L}\varvec{n}\:\varvec{K}}_{\varvec{d}}\:=-\left(\frac{\varDelta\:\varvec{H}}{\varvec{R}}\right)\frac{1}{\varvec{T}}+\frac{\varDelta\:\varvec{S}}{\varvec{R}}$$

Where K_d_ is the distribution coefficient (dimensionless), R (8.314 J/mol K) is the universal gas constant, and T (kelvin) is the absolute temperature. C_ads_, C_o_ and C_e_ are the concentrations of adsorbed AR8 dye at equilibrium, concentration of initial AR8 dye in solution and concentration of AR8 dye in solution at equilibrium (mg/L) respectively.

## Results and discussion

### Evaluation the %G and %GE of XG-g-PAm hydrogel

The results of %G and %GE are given in Table 1 which showed that the grafting process of Am on XG is marked by its effectiveness and simplicity, facilitated by the rapid heating provided by microwave technology, variety of reaction conditions^29^.

### Characterization

#### FTIR spectroscopy

**Fig. 4:**
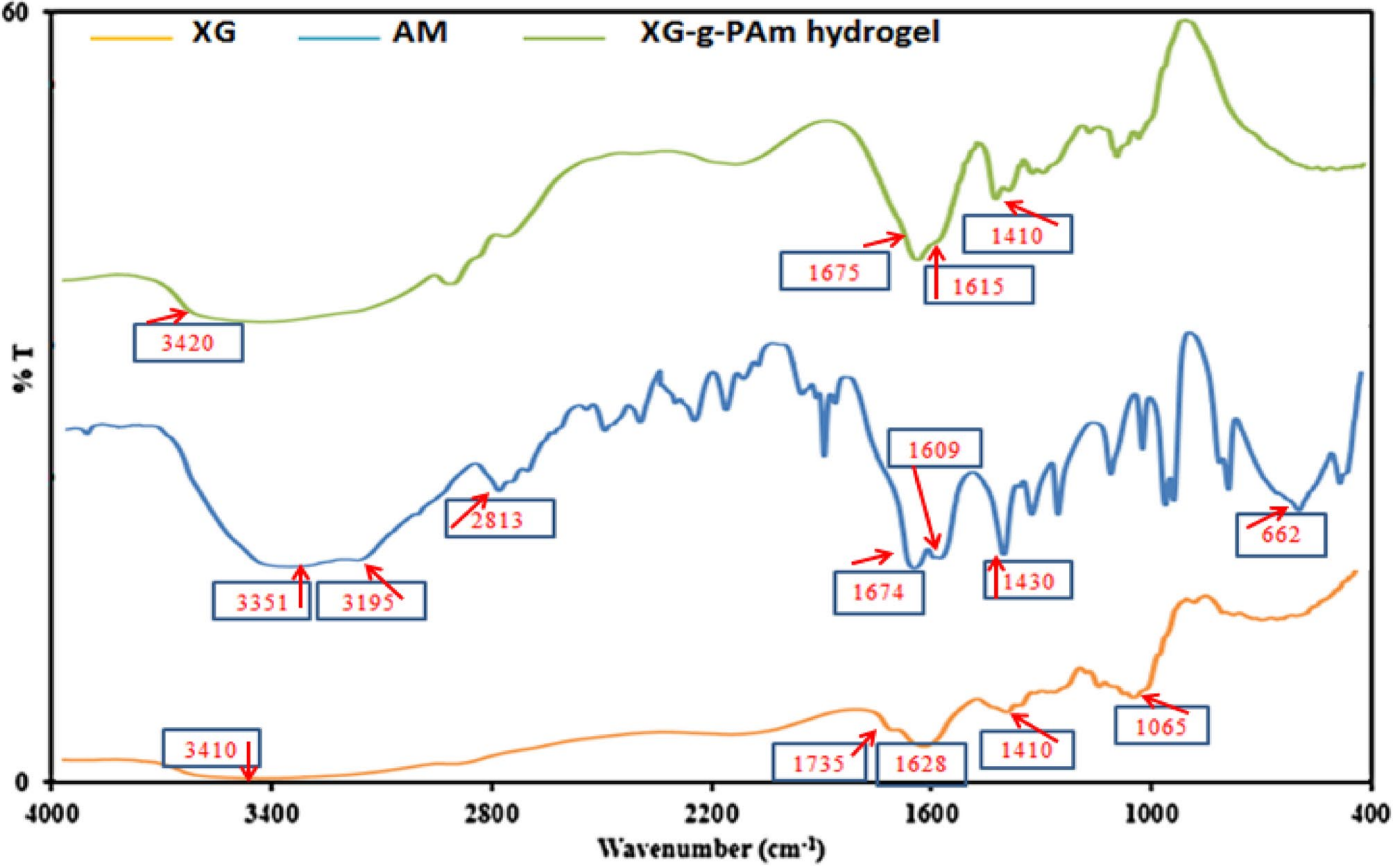
FTIR spectra of native XG, Am and XG-g-PAm 4 hydrogel.

FTIR spectra of native XG, Am and XG-g-PAm 4 hydrogel were illustrated in Fig. 4.

For native XG, the spectrum exhibits a distinct broad band at 3410 cm⁻¹, attributed to the vibrations of the hydroxyl group. The peaks at 1735 cm⁻¹ and 1628 cm⁻¹ are ascribed to the C = O vibrations of alkyl esters and the asymmetric stretching of carboxylate groups, respectively. Additional characteristic bands of XG observed at 1410 cm⁻¹ and 1065 cm⁻¹ are associated with C–H bending of the methyl group and C–O stretching of the C-OH group, respectively^30,31^.

For Am, the two bands at 3351 and 3195 cm^−1^ are due to stretching vibration of the amide functional group (NH_2_), the band at 2813 cm^−1^ is due to C-H stretches of CH_2_ group. The band at 1674 cm⁻¹ corresponds to the stretching vibration of C = O in the amide group, while the band at 1609 cm⁻¹ is associated with C = C stretching vibration, and the band at 1430 cm⁻¹ is linked to C–N stretching vibration^32^.

XG-g-PAm 4 hydrogel spectrum showed a combination of XG and Am absorption bands which indicates that the grafting process was successful. The broad band at 3420 cm⁻¹ results from the overlapping of the stretching vibrations of the N-H amide group of Am and the O-H group of XG. The peak at 1675 cm^−1^ is related to stretching vibration of C = O of amide group of acrylamide monomers, the disappearance of the peak at 1609 cm^−1^ which is related to stretching vibration of C = C of Am is considered as evidence of grafting occurrence. Furthermore, the broad absorption band at 1410 cm^−1^ is caused by the overlap of the C-H bending band of XG methyl group and the C-N stretching band of Am^31^.

### SEM characterization

**Fig. 5:**
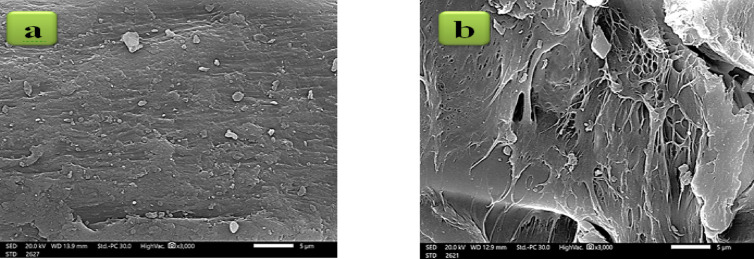
SEM of (**a**) Native XG and (**b**) XG-g-PAm 4 hydrogel.

Morphological appearance of XG-g-PAm 4 hydrogel had changed when compared to native XG sample. As observed in Fig. 5, the smooth surface of native XG became rougher when it was grafted with Am. XG-g-PAm 4 hydrogel showed a large number of pores and heterogeneous pore distribution^33^.

### TGA characterization

**Fig. 6:**
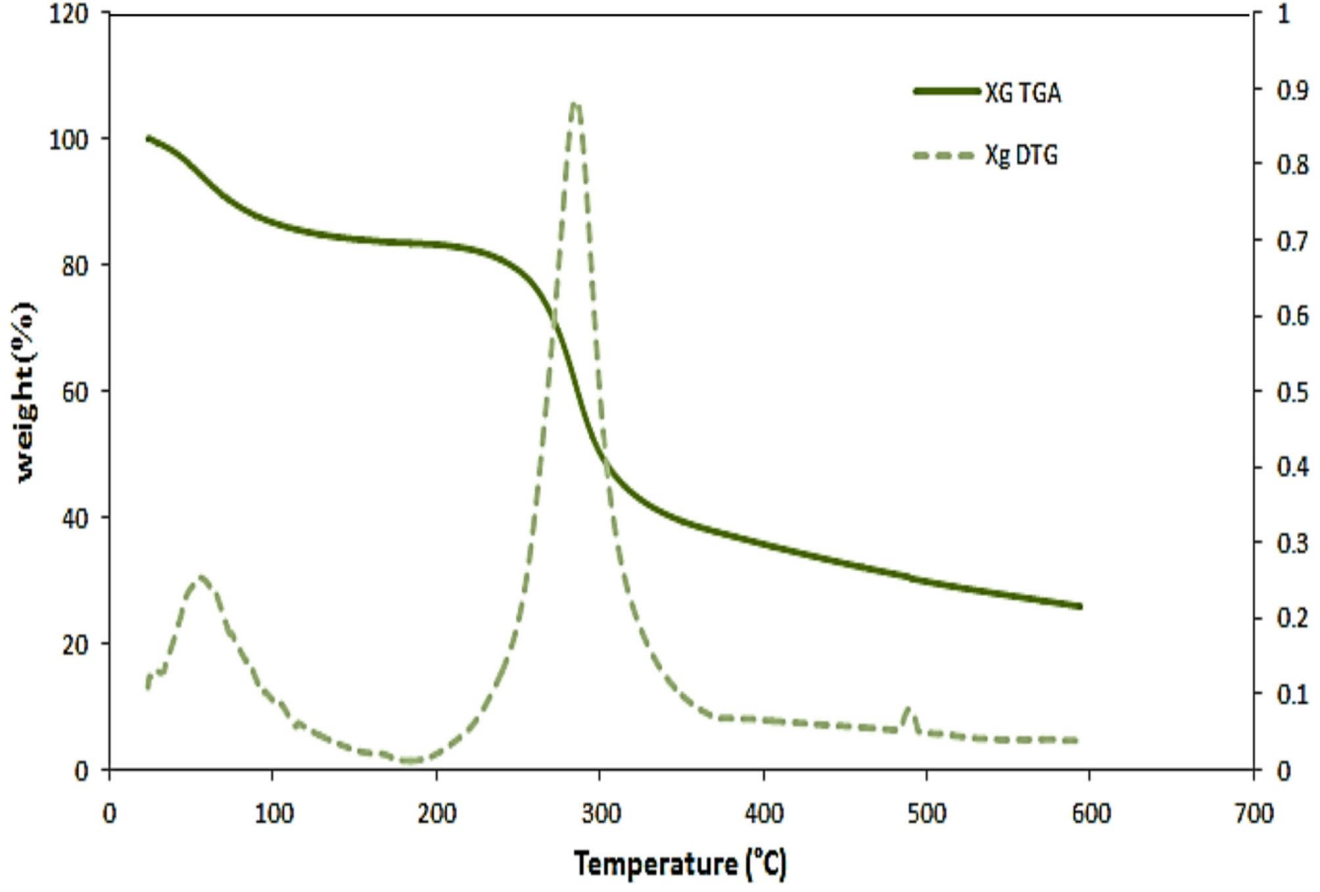
TGA and DTG of native XG.

Thermograms and deriavtograms revealed the thermal behavior of native and grafted XG, showing two stages of weight loss for native XG in Fig. 6. The first stage, from ambient temperature to 100 °C, resulted in a 13.3% weight loss likely due to moisture evaporation. The second stage, between 230 °C and 330 °C, showed a major weight loss of 41.7%, attributed to the degradation of the XG backbone. After these stages, the rate of weight loss decreased, leaving a residue of 25.9% at 600 °C.

**Fig. 7:**
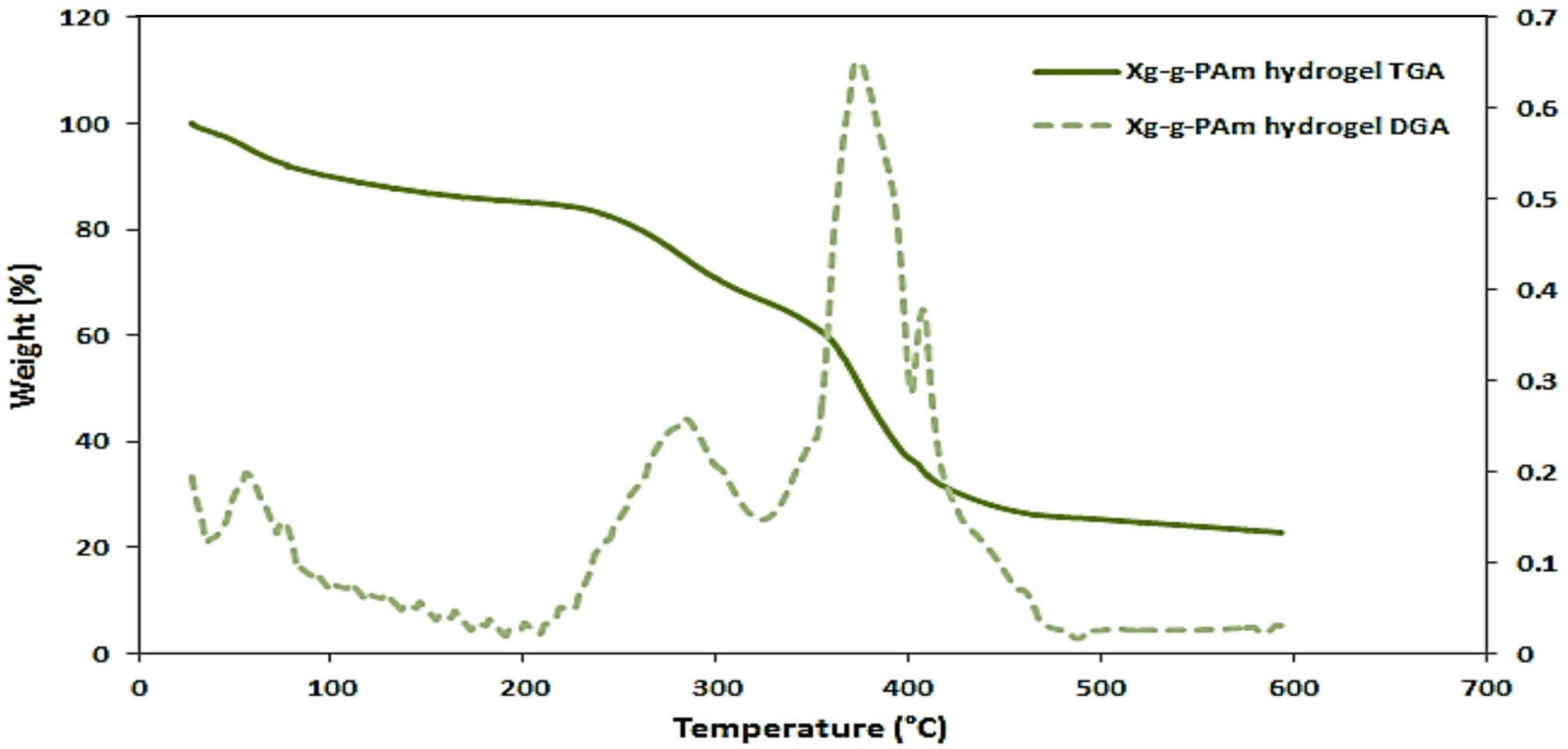
TGA and DTG of XG-g-PAm 4 hydrogel.

In case of XG-g-PAm 4 hydrogel (Fig. 7) it showed three stages of weight loss. The first stage started from ambient temperature and ended at 200 °C with initial weight loss of about 13.9% of the sample weight, is due to evaporation of adsorbed water by hydrogel. The difficulty in removing physically absorbed water from the 3D network of the hydrogel may explain the increase in final temperature at this point. The second stage took place within the range from 230 to 340 °C with a weight loss of 20.1% is due to degradation of XG backbone. The third stage began at 340 °C and ended at 500 °C, with a weight loss of approximately 38.7% owing to degradation of the polyacrylamide network, which was grafted on XG backbone and constructed a crosslinking three dimensional hydrogel network with XG. After 500 °C, decomposition was extremely slow, with a weight loss of about 2.5%.

**Fig. 8:**
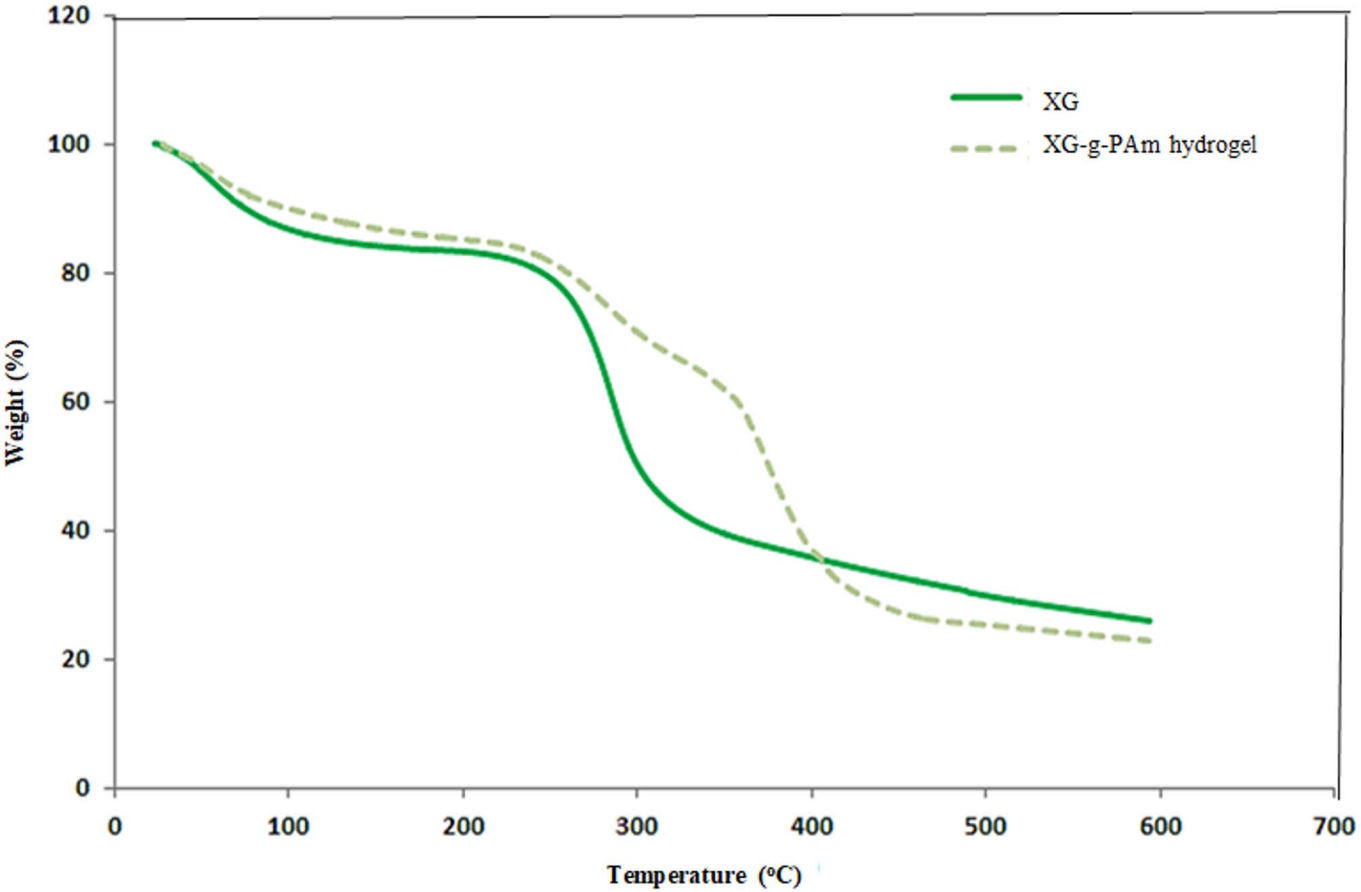
Comparative TGA thermograms of XG and XG-g-PAm 4 hydrogel.

The TGA thermogram comparison between XG and the hydrogel clearly indicated that grafting XG with PAm resulted in increased thermal stability for the hydrogel, Fig. 8.

### Interpretation of dye adsorption mechanism

Firstly, AR8 dye dissolved in aqueous solution then AR8 sulfonate groups (D-SO_3_Na) underwent dissociation and transformeds to anionic ions as mentioned in equation below^34,35^:

14 $$\mathbf{D-SO_3Na} \rightarrow \mathbf{H_2O\: D-SO_3^- + Na^+}$$

In the presence of excess H⁺ ions, the acrylamino groups grafted onto the xanthan gum backbone (R-CONH₂) and the hydroxyl groups (R-OH) became protonated.

15 $$\mathbf{R-CONH_2 + H^+ \rightleftharpoons R-CONH_3^+}$$

16 $$\mathbf{R-OH + H^+ \rightleftharpoons R-OH_2^+}$$

The adsorption process progresses through electrostatic interactions between the ions in the XG-g-PAm hydrogel and the dye, facilitated by the counterion reaction.

17 $$\mathbf{{}^+H_2O-R-CONH_3^+ + Dye-SO_3^-\rightleftharpoons Dye-SO_3^- + H_2O-R-CONH_3^+ -O_3S-Dye}$$

### Swelling study

**Fig. 9:**
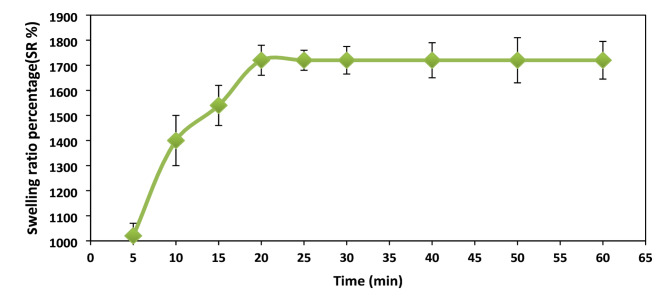
Swelling ratio percentage (% SR) of XG-g-PAm 4 hydrogel in deionized water at pH = 1.

The swelling behavior of XG-g-PAm 4 hydrogel at different time intervals using deionized water adjusted at pH = 1 was represented in Fig. 9. Initially the XG-g-PAm hydrogel’s swelling ratio percentage raised with time until reached to the equilibrium value which means that the water uptake has not increased over time beyond this point. The maximum swelling ratio percentage was found to be 1720% after 20 min.

The swelling was occurred due to protonation of acryl amino groups (CONH_2_) of acrylamide which cause the identical charged ions repelling one another electrostatically. The achieved equilibrium may results from the XG-g-PAm hydrogel’s pores being fully occupied, which prevents more water molecules from entering its network^36^.

### Point of zero charge

**Fig. 10:**
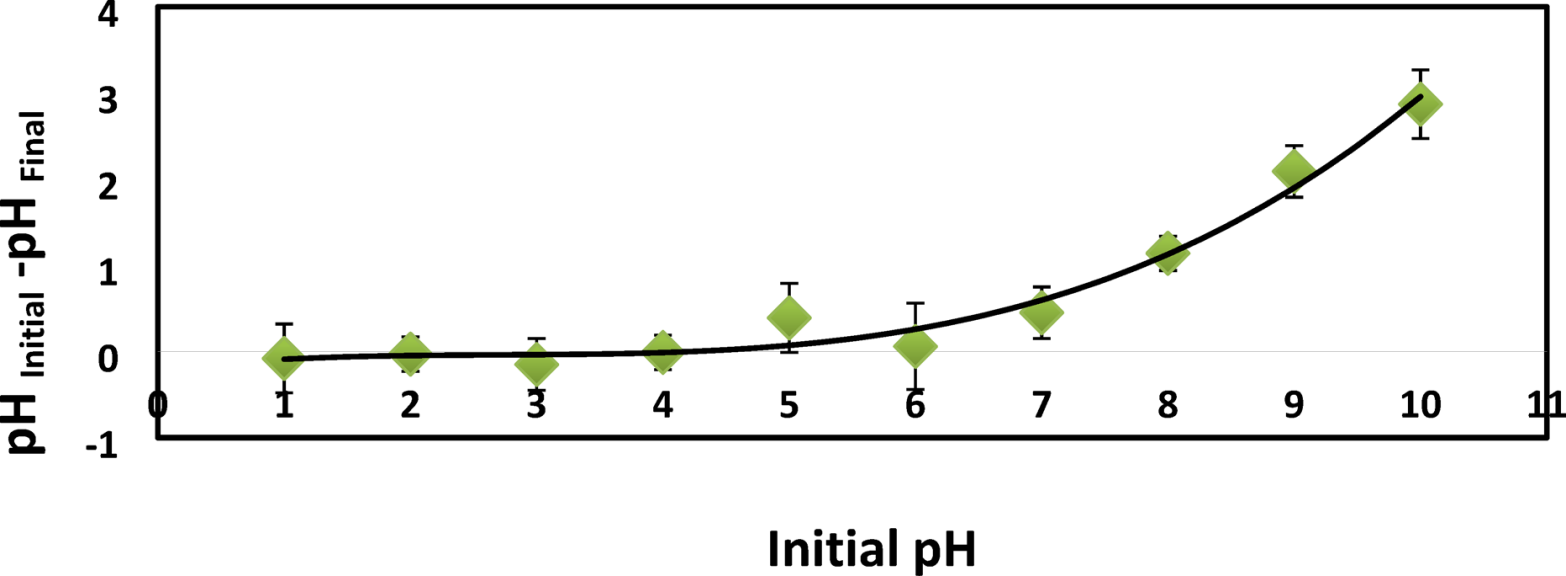
Determining the pHpzc of XG-g-PAm 4 hydrogel using the pH drift technique.

Figure 10 represent initial pH against ∆pH. The pH_PZC_ was found to be 4.3 which means that the surface of hydrogel is neutrally charged. Below this pH, the hydrogel surface becomes positively charged, while above it the surface become negatively charged.

### Effect of %G on AR8 adsorption

The effect of %G was carried out while other parameters were kept constant (adsorbent dose 0.05 g, pH = 1, initial dye concentration 500 mg/L, Contact time 2 h, Temperature 20 **±** 2 °C).

**Fig. 11:**
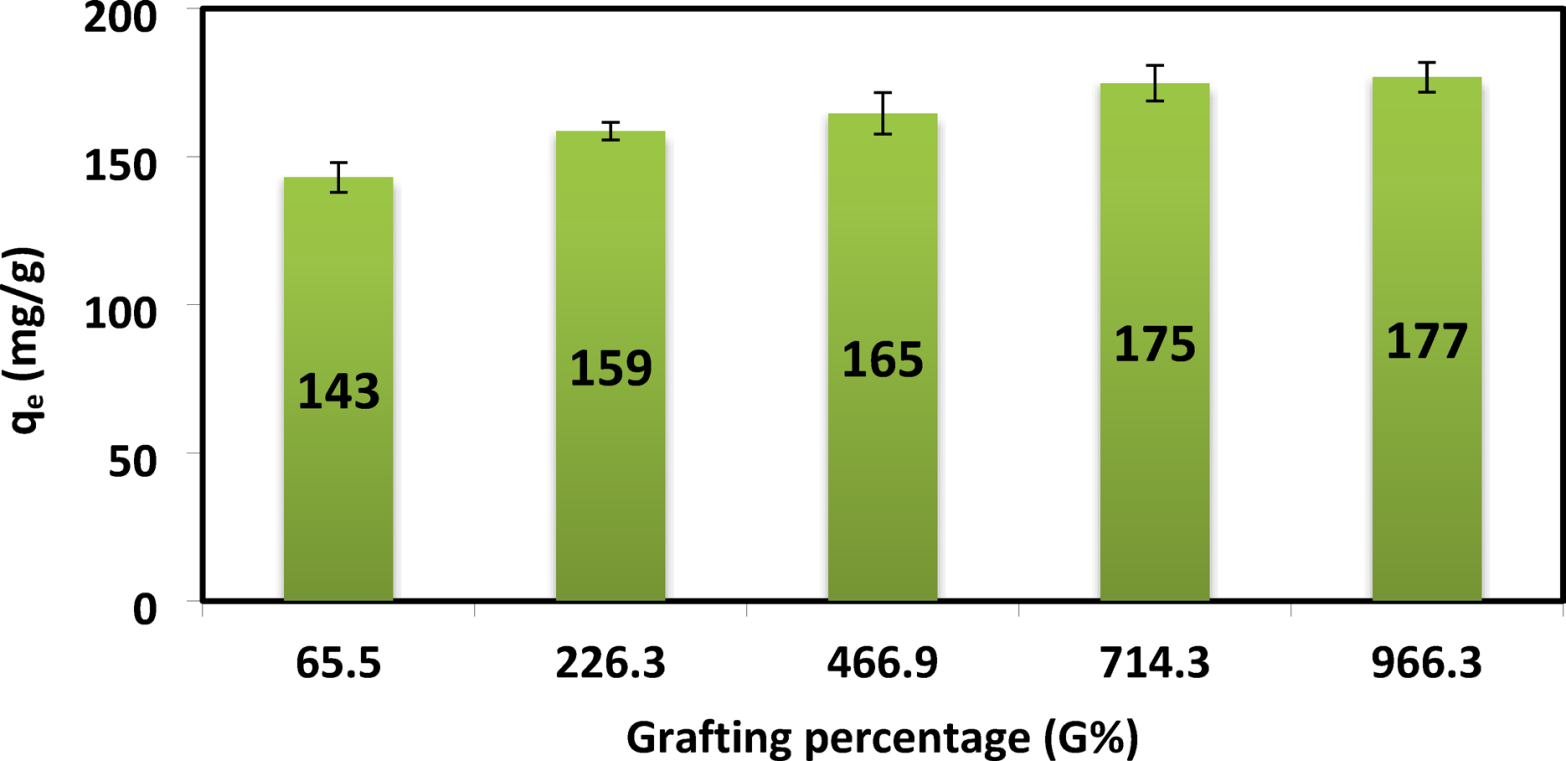
Effect of % G of XG-g-PAm 4 hydrogel on the adsorption capacity (q_e_) of AR8 dye (mg/g).

Acrylamide monomers were grafted onto the XG backbone to improve the hydrogel’s adsorption properties by increasing functional groups for binding AR8 dye anions. This led to an increase in q_e_ from 143 to 177 mg/g with higher grafting percentages. The additional polyacrylamide grafts enhanced surface polarity and hydrophilicity, thus improving dye adsorption (Fig. 11)^14,37^.

### Effect of pH on AR8 adsorption

The effect of pH was carried out while other parameters were kept constant (adsorbent dose [XG-g-PAm 4] 0.05 g, initial dye concentration 500 mg/L, Contact time 2 h, Temperature 20 **±** 2 °C).

**Fig. 12:**
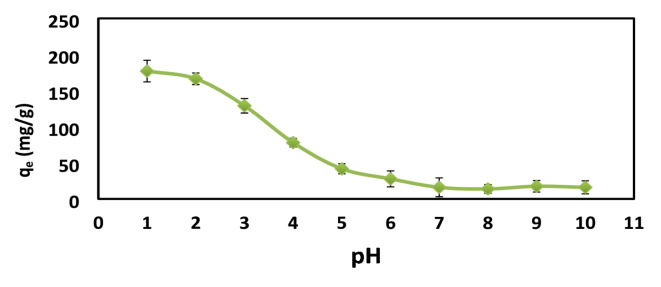
Effect of pH on the adsorption capacity (q_e_) of XG-g-PAm 4 hydrogel for AR8 dye.

The pH of the AR8 dye solution significantly affects the functional hydroxyl and acryl amino groups found in XG-g-PAm hydrogel, a multifunctional adsorbent. As seen in Fig. 12, The adsorption capacity (q_e_​) of AR8 dye decreases from 177 mg/g to 16 mg/g as the pH increases from 1 to 10 due to the electrostatic interaction between the anionic dye and the positively charged hydrogel surface^38,39^. The PZC of the hydrogel is 4.3. In acidic conditions (pH < pH_PZC_), more protons are available to protonate the hydroxyl and acryl amino groups, increasing dye adsorption through enhanced electrostatic attraction. In basic conditions (pH > pH_PZC_) the sorbent has a negative charge (which electrostatically repels anionic dyes and this inhibits their sorption)^40,42^.

### Effect of contact time

**Fig. 13:**
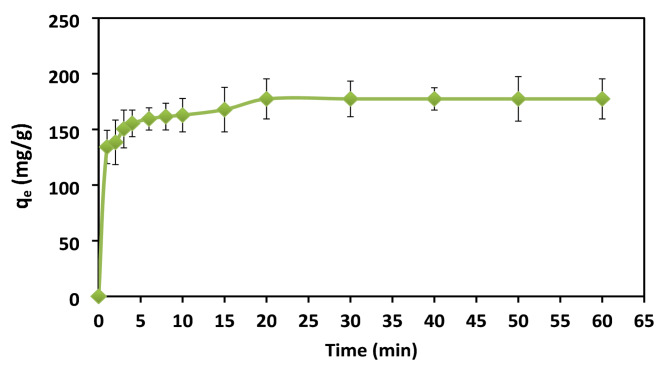
Effect of contact time on adsorption capacity (q_e_) of AR8 dye onto XG-g-PAm 4 hydrogel.

The effect of Contact time was carried out while other parameters were kept constant (adsorbent dose [XG-g-PAm 4] 0.05 g, initial dye concentration 500 mg/L, pH = 1, Temperature 20 ± 2 °C). Contact time is a highly significant parameter in the design and evaluation of adsorbent for dye removal from aqueous solutions. Figure 13 represented the effect of contact time on the q_e_of AR8 dye (mg/g) adsorbed onto XG-g-PAm hydrogel. It was noticed that the amount of adsorbed AR8 dye increased rapidly until equilibrium state was reached at 20 min (177 mg/g). The initial stage increased rapidly because of the greater availability of vacant sites causing an increase in the concentration gradient between adsorbate in solution and adsorbate on the surface of adsorbent which tend to accelerate the dye adsorption in the initial phase. This concentration gradient decreased over time due to accumulation of dye molecules in the vacant sites^43^^–^^45^. The q_e_ reached the equilibrium due to the saturation of the binding sites with AR8 dye molecules.

### Effect of dosage of XG-g-PAm hydrogel

The effect of hydrogel dosage was carried out while other parameters were kept constant of (contact time 2 h, XG-g-PAm 4, initial dye concentration 500 mg/L, pH = 1, Temperature 20 ± 2 °C).

**Fig. 14:**
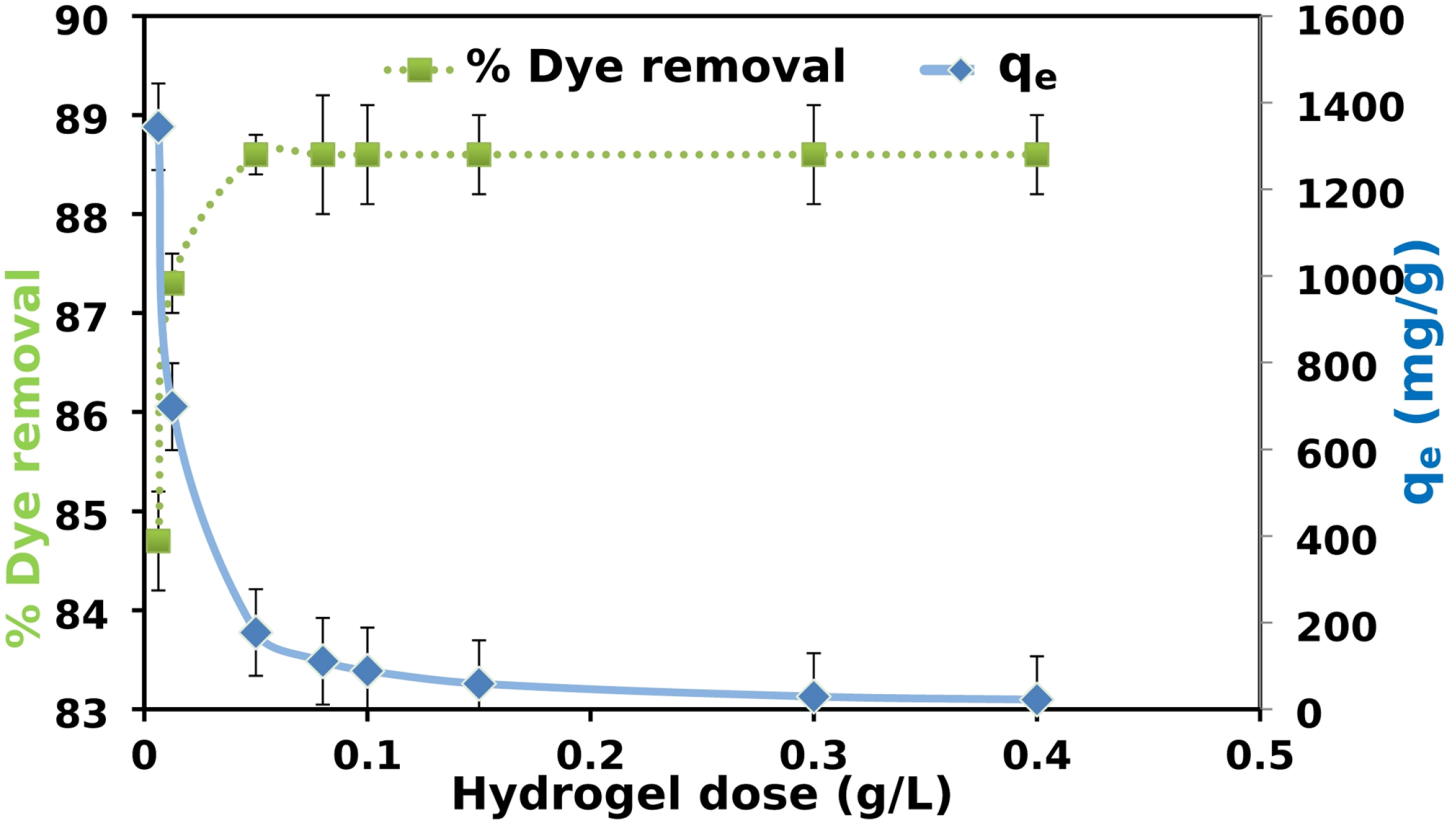
Effect of XG-g-PAm 4 hydrogel dosage (g/L) on the adsorption capacity (q_e_) (mg/g) and the dye removal (%).

The effect of XG-g-PAm hydrogel dosage on AR8 dye removal (%) and q_e_ was illustrated in Fig. 14. The percentage of dye removal (%R) improved from approximately 84.7 to 88.6% when the hydrogel dose raised from 0.0063 to 0.05 g/L. Beyond 0.05 g/L, the %R remained nearly constant, indicating that 0.05 g/L was the optimal dose for maximum dye removal. The initial increase in dye removal is likely due to the increased availability of the hydrogel’s exchangeable sites^32^.

The results showed also that, the q_e_ of AR8 decreased from 1344 to 22 mg/g with increasing the hydrogel dose from 0.0063 to 0.4 g/L due to decreasing the amount of dye adsorbed per unit mass of adsorbent. On the other hand, the percent of dye removal increases with increasing the adsorbent dose from 0.0063 to 0.05 g/L, further increase in adsorbent dose over 0.05 g/L, shows no increase in the percent dye removal due to adsorbent particle aggregation, which in turn caused a decrease in the total XG-g-PAm hydrogel surface area available for AR8 dye molecules and an increase in diffusion path length^32,46,47^.

### Effect of initial AR8 dye concentration

**Fig. 15:**
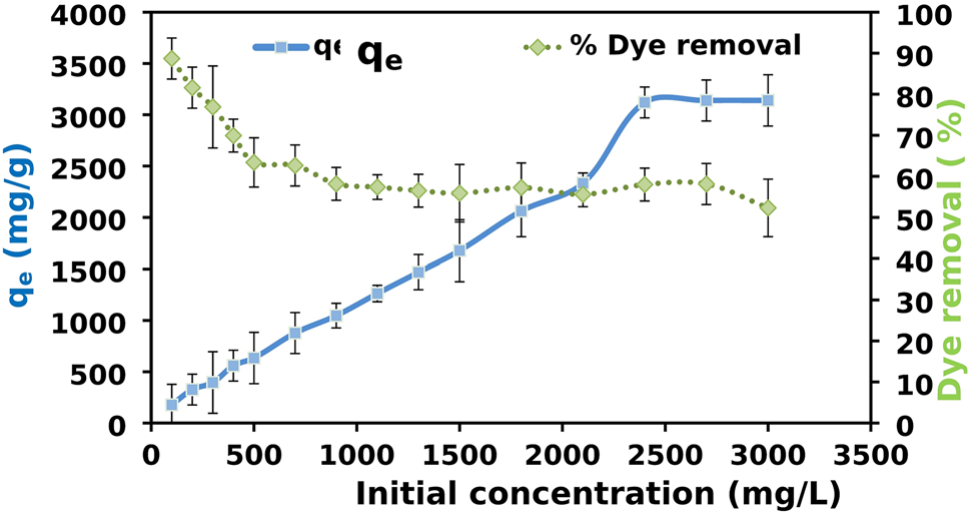
Effect of initial AR8 dye concentration on AR8 dye removal (%) and adsorption capacity (q_e_) of AR8 dye onto XG-g-PAm 4 hydrogel.

The effect of initial dye concentration was carried out while other parameters were kept constant of (contact time 2 h, adsorbent dose [XG-g-PAm 4] 0.05 g, pH = 1, Temperature 20 ± 2 °C). Figure 15 shows that as the initial AR8 dye concentration increases from 100 to 3000 mg/L, the dye removal efficiency decreases from 88.7–52.3% due to limited active sites on the hydrogel surface, causing competition among dye molecules at higher concentrations. Conversely, the adsorption capacity (q_e_) of AR8 increases from 177 to 3140 mg/g with higher dye concentrations, driven by an enhanced diffusion process overcoming mass transfer resistance. However, beyond 2400 mg/L, q_e_ plateaus, indicating the saturation of adsorption sites on the XG-g-PAm adsorbent.

### Effect of temperature

The effect of Temperature was carried out while other parameters were kept constant of (contact time 2 h, adsorbent dose [XG-g-PAm 4] 0.05 g, pH = 1, initial dye concentration 500 mg/L).

**Fig. 16:**
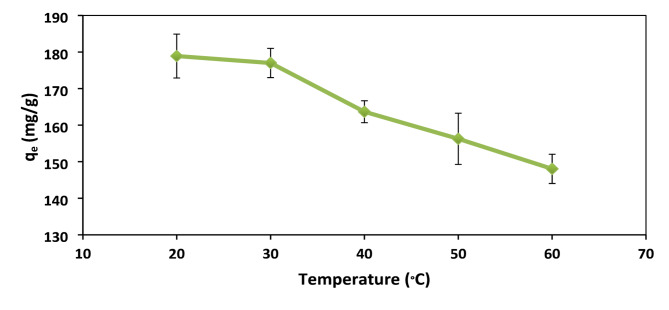
Effect of temperature on adsorption capacity (q_e_) of AR8 dye onto XG-g-PAm 4. hydrogel.

Temperature is a crucial factor in the dye adsorption process, indicating whether it is exothermic or endothermic. As shown in Fig. 16, increasing the temperature from 20 to 60 °C reduces the %R from 88.6 to 74.5% and the q_e_ from 178 to 147 mg/g. This decrease suggests an exothermic adsorption process, likely due to weakened electrostatic interactions, increased dye molecule mobility, and the shrinking of the hydrogel’s polymeric units, all of which reduce the hydrogel’s swelling ability.

### Adsorption kinetics

Plots of log (q_e_ − q_t_) vs. time and t/q_t_ vs. time were constructed. The slope and intercept of these plots were used to calculate theoretical values of q_e_ and k_1_ for the pseudo-first-order model (Fig. 1S), and q_e_ and k_2_ for the pseudo-second-order kinetic model (Fig. 2S).

All parameters (k_1_, q_e_, k_2_) and corresponding linear regression correlation coefficient (R^2^ values of pseudo-first order and pseudo-second order were listed in Table 2.

**Table 2:** Pseudo-first order and pseudo-second order kinetic models’ parameters for adsorption of AR8 dye onto XG-g-PAm 4 hydrogel.

Adsorbent	q_e_(exp) (mg/g)	Pseudo first order kinetic model			Pseudo second order kinetic model		
		k_1_ (min^−1^)	q_e_ (cal) (mg/g)	R^2^	k_2_ (g/mg.min)	q_e_ (cal) (mg/g)	R^2^
XG-g-PAm 4	177	0.147	60	0.6945	9.80ₓ10^−3^	179	0.9998

It was noted that R^2^ of pseudo-second order (0.9998) which was greater than that of the pseudo-first order (0.6945). Also, the significant differences between the experimental (177 mg/g) and the calculated equilibrium adsorption capacity (60 mg/g) of pseudo-first order confirmed that the experimental data don’t obey the pseudo-first order.

The highest R^2^ of pseudo-second order (0.9998) and the compatibility of the calculated q_e_ (177 mg/g) with the experimental one which was (179 mg/g) proved that the adsorption of AR8 dye onto XG-g-PAm 4 hydrogel obeys pseudo-second order model.

### Adsorption isotherms

**Fig. 17:**
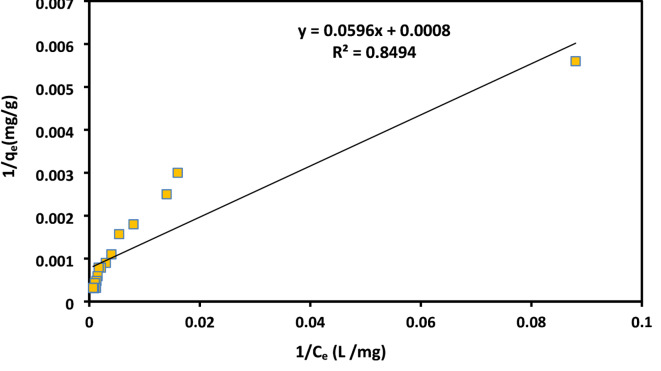
Langmuir isotherm of AR8 dye adsorbed onto XG-g-PAm 4 hydrogel.

**Fig. 18:**
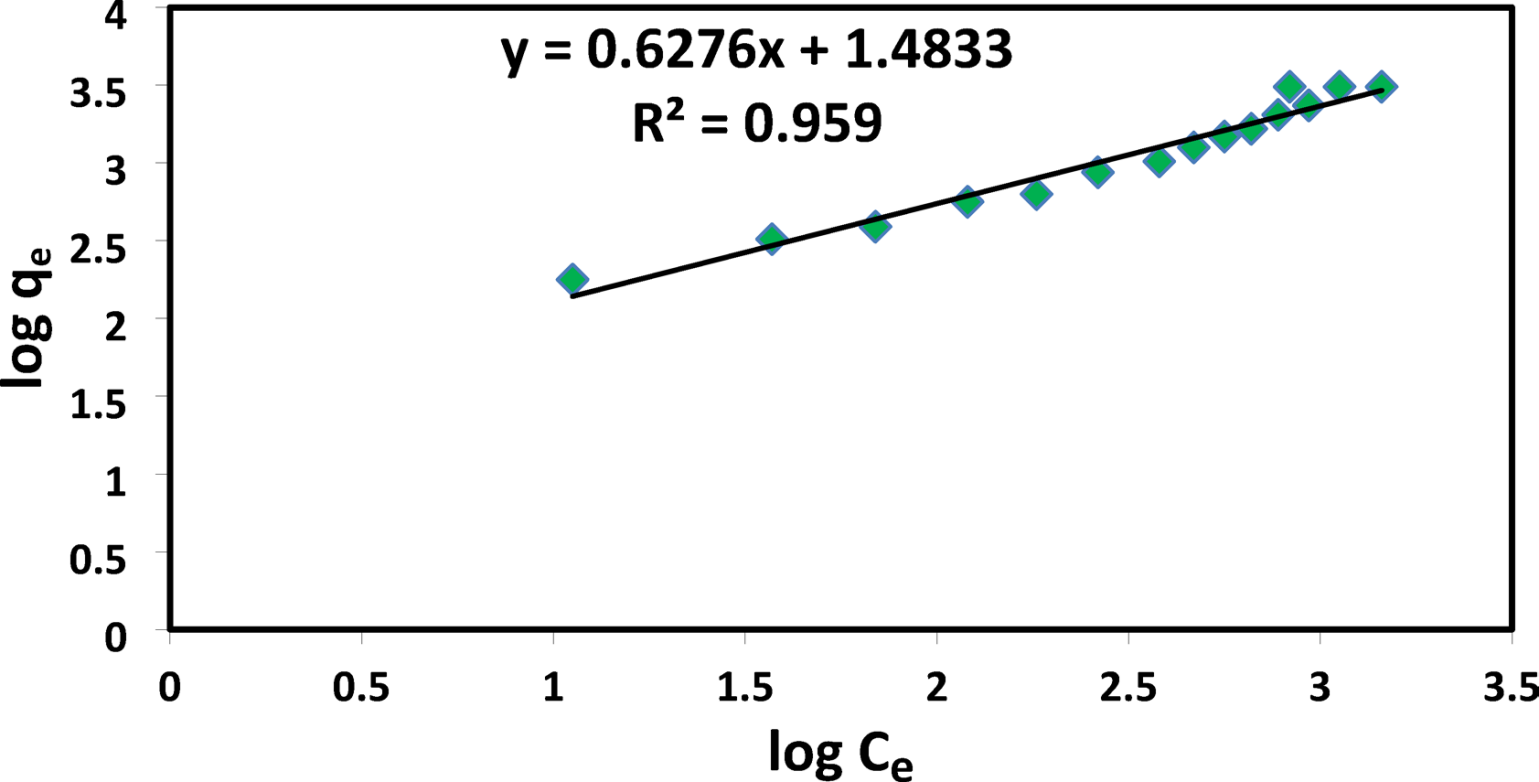
Freundlich isotherm of AR8 dye adsorbed onto XG-g-PAm 4 hydrogel.

The linear fitting plots for the Langmuir and Freundlich isotherm models for AR8 dye adsorption on XG-g-PAm 4 hydrogel are provided in Figs. (17 and 18). The isotherm coefficients and parameters derived from these equilibrium plots are summarized in Table 3.

**Table 3:** Isotherm parameters obtained from Langmuir and Freundlich equations.

**Langmuir**	**Q**_o_ (mg/g)	**K**_L_ (L/mg)	**R** ^2^
**Parameter**	1250	0.0134	0.849
**Freundlich **	**1/n**	**K**_**f**_ **(mg/g) (L/mg)**^**1/n**^	**R** ^**2**^
**Parameter**	0.6276	30.43	0.959

Table 3 showed that R^2^ of Freundlich isotherm (0.959) was higher than that of Langmuir isotherm (0.849) which means that the experimental results were fitted well with Freundlich model. This means that heterogeneous sorption sites form the majority of the adsorbent surface while homogeneous sorption sites are fewer. the value of *n* > 1 which means that the adsorption of AR8 dye onto XG-g-PAm hydrogel is physical adsorption^48^.

### Adsorption thermodynamics

The calculated values for the changes of Δ*G*°, Δ*H*°, and Δ*S*° are shown in Table 4.

**Table 4:** Thermodynamic data for AR8 dye adsorption onto XG-g-PAm 4 hydrogel at different temperatures.

Temperature (K)	ΔG (k J/mole)	ΔH (J/mole)	ΔS (J/mole K)
293	−18.74	−24.13	−63.89
303	−19.38		
313	−20.03		
323	−20.66		
333	−21.29		

The negative value of ΔG in Table 4 indicates that AR8 dye adsorption onto XG-g-PAm hydrogel was spontaneous. Adsorption is physisorption when the values of ΔG ranged from − 20 to 0 k J/mole. while, chemisorption occurred when the values of ΔG fall between − 80 and − 400 k J/mole^49^. The obtained values of ΔG were found to be in the range of −18.74 to −21.29 k J/mole, indicating that the adsorption of AR8 dye onto XG-g-PAm hydrogel was physisorption.

The negative sign of ΔH and ΔS show that the adsorption of AR8 dye onto XG-g-PAm hydrogel was an exothermic process, and the system’s randomness reduced near solid-liquid interfaces^50,51^.

## Reusability study

The effectiveness of an adsorbent largely depends on its reusability and durability. To assess the reusability of the hydrogel, five cycles of adsorption-desorption studies were carried out. Following adsorption, desorption was performed by immersing the dye-loaded hydrogels in a solution with a pH of approximately 10.The hydrogels were then neutralized with a 1 M HCl solution and rinsed with distilled water before starting the next adsorption cycle^15,48,52^.

**Fig. 19:**
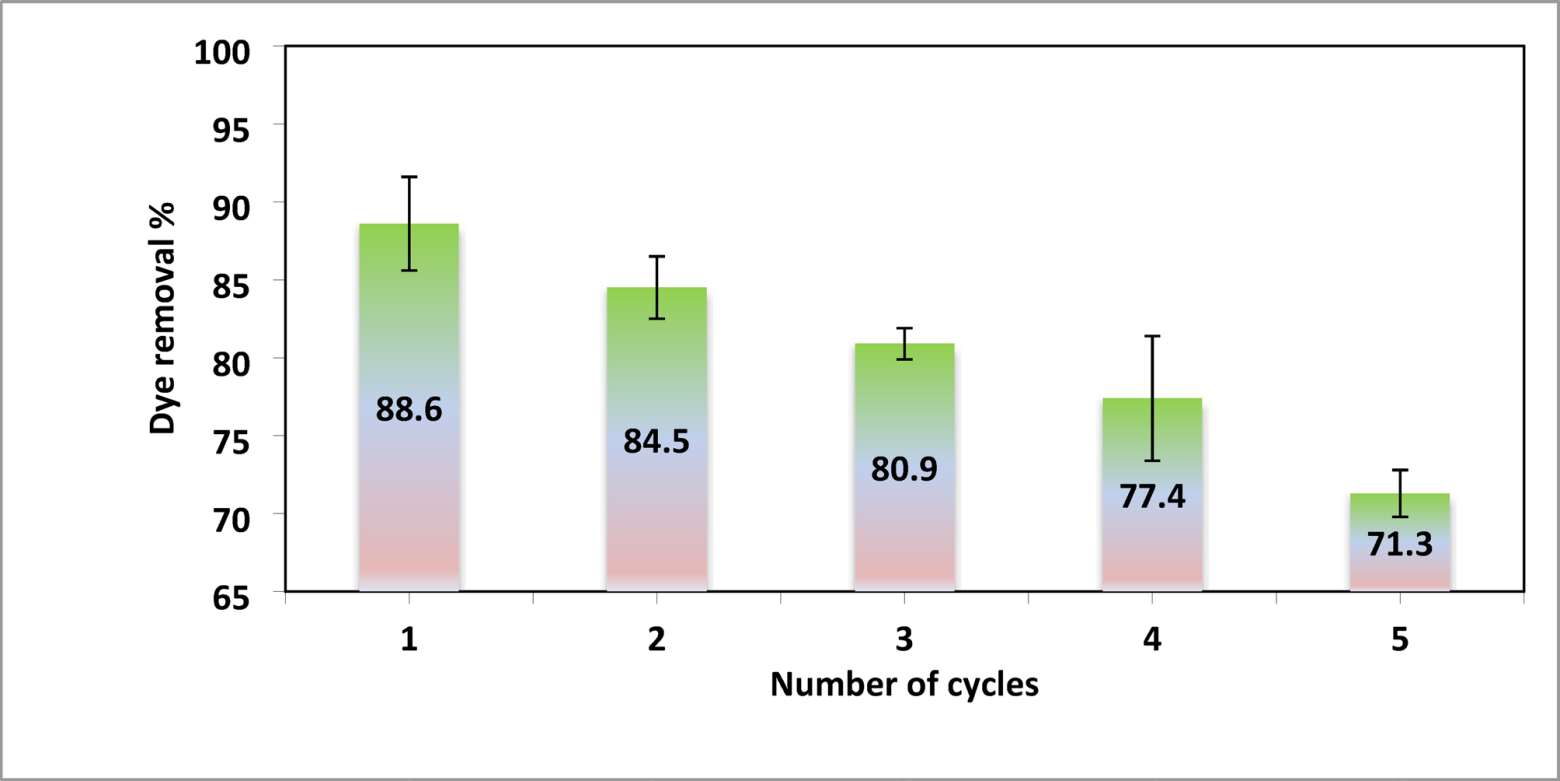
Reusability of XG-g-PAm 4 hydrogel for removal of AR8 dye.

Figure 19 depicted AR8 dye removal (%) onto XG-g-PAm 4 hydrogel for five successive adsorption-desorption cycles. The dye removal (%) decreased from 88.6 to 71.3% after the fifth cycle due to the possibility of polymer chain breakage as a result of the repeated base treatment during the reusability process. Thus, the studied results showed that XG-g-PAm hydrogel maintains well its adsorptive properties even after five successive cycles.

## Comparative study

Table 5 shows a comparison between the adsorption capacity (q_e_) of XG-g-PAm hydrogel and other previously reported adsorbents for removing of AR8 dye in recent years.

**Table 5:** Comparison of adsorption capacity (q_e_) of different adsorbents for removing of AR8 dye.

Adsorbents	Maximum adsorption capacity Q_o_ (mg/g)	Reference
XG-g-PAm hydrogel	1250	Present study
Guar gum grafted poly acrylamide hydrogel	54	(Batouti et al., 2022)^14^
Starch-g-poly (N, N-dimethyl acrylamide) hydrogel	120	(Sadik et al., 2020)^15^
Cellulose nanocrystal hydrogel	17	(Abdelaziz et al., 2022)^22^

## Conclusion

XG-g-PAm hydrogel was successfully synthesized by grafting acrylamide monomers onto XG polysaccharide in the presence of MBA as a crosslinker and KPS as an initiator using microwave assisted method. FTIR, SEM and TGA techniques were used to confirm the grafting reaction. At pH 4.3 the sorbent surface had a neutral charge. adsorption studies validate the initial Assumption that the prepared hydrogel has a notable adsorption capacity for anionic dyes like AR8, the adsorption capacity (q_e_) of AR8 dye onto XG-g-PAm hydrogel increased with higher grafting percentages, initial dye concentrations, and was optimized at a hydrogel dosage of 0.05 g/L and a pH of 1.The adsorption capacity decreased with rising temperatures, This finding is confirmed from the negative value of ∆H_ads_. The adsorption data aligns well with the Freundlich adsorption model which assume the heterogeneous adsorption with multilayers. Furthermore, the pseudo-second-order model showed the best alignment with the kinetic studies. From the reusability study it was found that the prepared hydrogel still had good adsorption properties, without any loss of structural stability even after five cycles with dye removal (%) of 71.3.

## Supplementary Information

Below is the link to the electronic supplementary material.

Supplementary Material 1

## Data Availability

The data will be available from the corresponding author upon request.
