# Supplementary figures and images for: Microwave-assisted synthesis and characterization of Xanthan gum-grafted polyacrylamide hydrogel for the removal of acid red 8 dye from aqueous solutions
Source: Sci Rep. 2025 Sep 12;15:32425. doi: 10.1038/s41598-025-14539-2 (PMC12432242; doi:10.1038/s41598-025-14539-2)

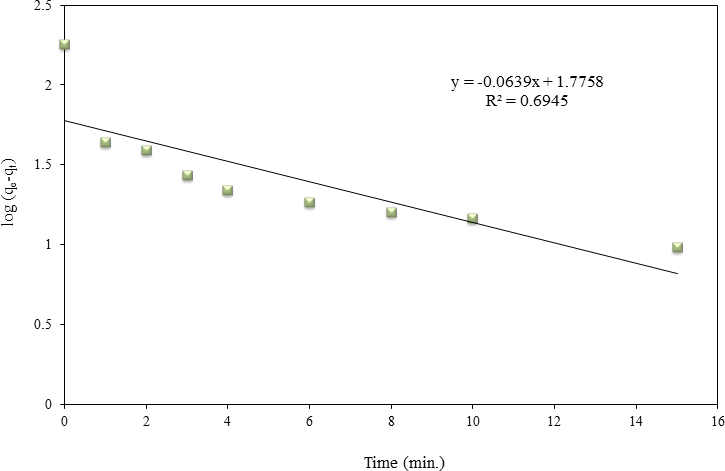


**Figure 1S:** Showed the plot of log (qe-qt) against t


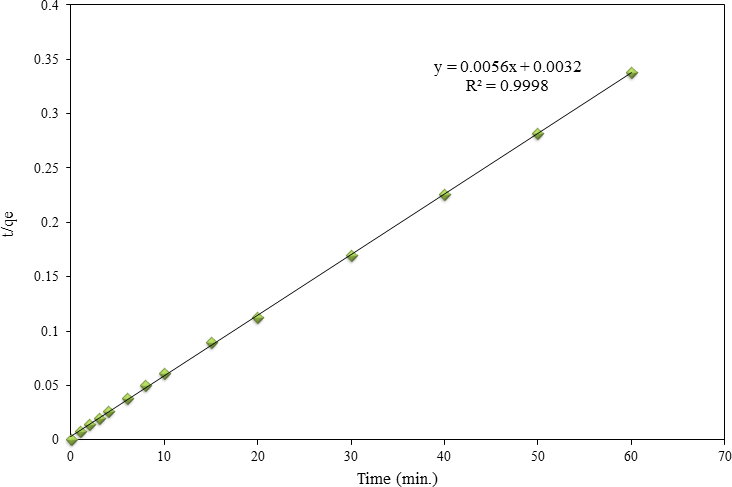


**Figure 2S:** Showed the plot of t/qt against t

Supplement: Supplementary file 1 — Supplementary Material 1 [file 41598_2025_14539_MOESM1_ESM.docx]
